# Effect of dental pain and caries on the quality of life of Brazilian preschool children

**DOI:** 10.11606/S1518-8787.2018052000093

**Published:** 2018-03-14

**Authors:** Maria do Carmo Matias Freire, Patrícia Corrêa-Faria, Luciane Rezende Costa

**Affiliations:** I Universidade Federal de Goiás. Faculdade de Odontologia. Departamento de Saúde Oral. Goiânia, GO, Brasil; II Universidade Federal de Goiás. Faculdade de Odontologia. Programa de Pós-Graduação em Odontologia. Goiânia, GO, Brasil

**Keywords:** Child, Preschool, Toothache, epidemiology, Dental Caries, epidemiology, Socioeconomic Factors, Quality of Life, Dental Health Surveys

## Abstract

**OBJECTIVE:**

To investigate the impact of dental pain on daily performances among five-year-old Brazilian children.

**METHODS:**

The study used data of 7,280 five-year-old children participating in the 2010 Brazilian Oral Health Survey (SBBrasil 2010 Project). Children were clinically examined and their parents or carers were interviewed at their homes. The outcome was the prevalence of the oral impacts on daily performance, and the explanatory variable was dental pain in the last six months. Other independent variables were children’s gender and skin color/race, family income, household overcrowding, and caries experience (dmft). Rao-Scott test and Poisson regression for complex samples were carried out.

**RESULTS:**

The prevalence of impacts on daily performances was 26.1% (95%CI 22.3–30.2). Significant associations were found between the outcome and pain, caries experience, and sociodemographic variables. After adjusting for the independent variables, only pain and caries remained significant. Impacts on daily performances were more frequent among children with pain (PR = 1.14, 95%CI 1.06–1.23) compared to those without pain. Children with low dmft (PR = 1.90, 95%CI 1.39–2.60) and those with high dmft (PR = 3.53, 95%CI 2.78–4.49) had a higher prevalence of impact than those with no caries experience.

**CONCLUSIONS:**

Dental pain and caries had strong negative impacts on the five-year-old children’s daily performances regardless of their demographic and socioeconomic characteristics.

## INTRODUCTION

Assessment of the psychological well-being in addition to clinical evaluation has been proposed to provide a better understanding of the impact of oral health problems on people’s lives ^1^ . The sociodental approach has attracted growing interest and in recent years several studies have been carried out on oral health-related quality of life (OHRQoL). While the impact of oral diseases, mainly dental caries, has been investigated in preschool children ^2–10^ , there are currently few studies on the impact of dental pain in this age group.

Dental pain prevalence in preschool children varies from approximately 7.0% to 22.0% ^3,11–16^ , and given its impact on children and families as well as the possibility of it being preventable and treated, it can be seen as a major public health problem. Differences in the prevalence rates reported are due to diverse study designs and instruments used to collect data, as assessing pain in young children is a difficult task even for experienced dentists and it is extremely dependent on parental or carers’ perceptions.

Dental caries and dental pain are strongly related ^11^ ^,^ ^12^ ^,^ ^15^ , although not all children with caries experience report pain or any impact on their oral health-related quality of life (OHRQoL) ^14^ ^,^ ^17^ . A birth cohort study with 1,129 five-year-old children showed a prevalence ratio of 4.8 (95%CI 3.3–7.1) for children with high caries occurrence reporting dental pain ^13^ . In other studies with three- to five-year-olds attending preschools, pain but not caries was associated with a negative impact on OHRQoL, especially on eating and drinking, whilst high-severity caries negatively impacted speech ^3^ ^,^ ^18^ .

The aforementioned studies assessed the impact of dental caries/pain on the OHRQoL in non-representative samples of children seeking dental treatment ^2^ , attending nurseries ^6^ or participating in immunization campaigns ^8^ ^,^ ^11^ , or explored OHRQoL in representative samples limited to specific regions ^4^ ^,^ ^5^ ^,^ ^7^ ^,^ ^9^ ^,^ ^10^ ^,^ ^12^ ^,^ ^18^ . To the best of our knowledge, there is no investigation about the impact of dental pain on the quality of life of preschool children in a large national data. In fact, the United Kingdom’s 2003 Children’s Dental Health Survey showed that the most frequently reported oral impact that parents are aware of in their children is pain, but they did not investigate in depth the association between dental pain and OHRQoL in five-year-old children ^14^ , which is also true for the 2013 survey ^19^ .

Results from national data can give a more comprehensive overview of the burden of dental diseases and strongly support the recommendation of policies that prioritize the solving of dental pain in young children. This is a frequently neglected age group in primary care and there is evidence that many children leave a dental consultation without receiving any oral procedure for pain relief ^20^ .

Studies based on the Brazilian 2010 National Survey of Oral Health data reported that dental pain and caries prevalence in five-year-old children were high and influenced by contextual and individual factors ^15^ ^,^ ^21^ . In the same survey, the Oral Impacts on Daily Performance (OIDP) was investigated for the first time, in an attempt to integrate psychological well-being measures with oral health surveillance. However, the association between quality of life, dental pain and dental caries in this group remain little explored.

A better understanding of the role of clinical and subjective measures of oral health on young children and their families’ quality of life is therefore needed. Previous studies focusing on representative samples of cities from different countries have included children attending preschools or immunization campaigns ^2–12,18^ ; this may have excluded the most vulnerable population. Conclusions from a national survey can have more external validity than other representative samples from specific locations, and then provide relevant data for policies on dental care for preschool children. Based on the evidence from previous studies regarding OHRQoL in specific groups ^2–12,18^ , we have hypothesized that both dental caries and pain strongly affect the daily performances of this age group. Results may lead to more appropriate health promotion measures towards reducing the burden of caries and its consequences in the primary dentition, which has generally received little attention worldwide. They may be also useful to evaluate oral health programs addressed to young children.

The objective of this study was to investigate the impact of dental pain on daily performances among five-year-old Brazilian children, using national epidemiologic data.

## METHODS

### Study Design and Sample

The data for this cross-sectional study were extracted from the database of the 2010 Brazilian Oral Health Survey conducted by the Ministry of Health and approved by the National Research Ethics Board ^22^ . This survey is also in accordance with the World Medical Association Declaration of Helsinki. Children’s parents or carers were previously informed about the study and asked to sign a consent form.

The sample size calculation for the 2010 survey was based on dental caries levels and resulted in at least 8,000 five-year-old children. Participants were selected using a probabilistic cluster sampling in the 27 capital cities and a random sample of 150 country towns of the five Brazilian regions. More detailed information on the National Survey has been presented elsewhere ^15^ ^,^ ^22^ .

### Data Collection

Teams constituted by a previously calibrated examiner and a recorder collected data through interviews and oral examinations at the selected families’ residences.

The dependent variable of this study was the parents’ or carers’ perceptions of their children’s oral health impact in their lives in the past six months as assessed by an abbreviated format of the OIDP. This OHRQoL indicator was initially developed for adults ^23^ and a validated Brazilian version is not yet available, although it has been extensively used. Due to the difficulties inherent to large epidemiological surveys, the same instrument was used for all age groups included (children, adolescents, adults and the elderly). For the five-year-old children, the following question regarding the OIDP was initially asked to the parents: “Some people have problems that can be caused by their teeth. Of the situations below, which have applied to your child in the last six months?” Questions included nine domains: eating and drinking, brushing teeth, feeling nervous, having leisure activities, practicing sports activities, speaking, smiling or talking, studying or doing school work, and sleeping. The response options were: “no”, “yes”, and “do not know or refused to answer”. The last was treated as missing information for each OIDP question. The participants who had presented at least one complaint were assigned to the category ‘impact on daily performance’, whereas the participants who had no complaints were assigned to the category ‘absence of impact’.

The explanatory independent variable was dental pain. Pain was evaluated through this question directed at the parent or carer: “Has your child felt dental pain in the last six months?” The response options were: “yes”, “no”, and “don’t know or can’t remember”. Only those who answered ‘yes’ or ‘no’ (n = 7,280) were included in the present analysis.

Caries experience in the deciduous teeth was evaluated according to the World Health Organization (WHO) criteria ^24^ . The following levels of decayed, missing, and filled teeth (dmft index) were considered for the analysis, based on tertiles of the frequency distribution: no caries (dmft = 0), low (dmft = 1 to 3) and high (dmft ≥ 4). Children were examined seated in front of the examiner under natural light, using plane mouth mirrors and WHO periodontal probes. The exams were performed by previously trained and calibrated dentists.

Other independent variables were demographic and socioeconomic: children’s gender and skin color/race, family income and household overcrowding. These were chosen because of their known association with dental caries and pain ^15^ ^,^ ^21^ . Sociodemographic factors were collected through a questionnaire answered by the parents or carers. The skin color/race was determined according to the criteria used in Brazil: white, black, *pardo* , yellow and indigenous ^25^ . Family income categories were based on the Brazilian currency and then converted to US dollars. The seven original categories for monthly family income in the original questionnaire were grouped by four, according to their frequencies, using a conversion rate of $1 US commercial dollar = $2.29 Brazilian Real (August 2014). Household overcrowding was the average number of dwellers per room used for sleeping in the children’s residences. Cut-off points for the last two variables were based on previous studies in the health field in Brazil ^26^ .

### Statistical Analysis

Descriptive analysis of the studied variables was initially performed using frequency distribution. The sample weights were then considered in the next steps, using the ‘Complex Samples’ module of the IBM SPSS Statistics software, version 21.0 (IBM Corporation, Armonk, New York, USA) and Stata software, version 13.0 (Stata Corp., College Station, TX, USA). The association between the OIDP prevalence and the independent variable was tested through the Rao-Scott test, which is a correction to chi-square tests for complex samples. Association between dental pain and caries was also tested and showed collinearity between them.

The next step was to investigate the association between the presence of impact (OIDP), dental pain and other independent variables (skin color/race and family income) using Poisson regression with a robust variance for complex samples. Those variables that were significant in the bivariate analysis were included in multiple regression models. The significance level was 5%. Additionally, the presence of an interaction between dental caries and dental pain was tested.

## RESULTS

A total of 7,348 children participated in this study (response rate = 91.8%). The children were predominantly boys (51.9%) and white (48.6%), and most of the families presented low income and high household overcrowding. Prevalence of dental pain was 22%. More than half of the children (54.1%) presented dental caries and 27.1% had high levels of the disease (dmft ≥ 4) ( Table 1 ). Mean dmft was 2.42 (SD = 3.36).

**Table 1:** Sociodemographic and clinical characteristics and reported dental pain of five-year-old children. Brazilian Oral Health Survey, 2010.

Variable	n ^a^	%
Gender (n = 7,348) ^a^		
Boys	3,673	51.9
Girls	3,675	48.1
Skin color/race (n = 7,348) ^a^		
White	3,290	48.6
Black	586	8.9
*Pardo*	3,274	39.2
Yellow (Asian descendants)	145	2.3
Indigenous	53	0.9
Family income (US$) (n = 7,002) ^a^		
≤ 218	1,537	21.8
219–656	3,754	52.9
657–1,093	1,034	17.0
≥ 1,094	677	8.3
Household overcrowding (n = 7,332) ^a^		
1 room or more/person	1,691	24.9
Less than 1 room/person	5,641	75.1
Dental pain (n = 7,280) ^a^		
No	5,760	78.0
Yes	1,520	22.0
Dental caries (n = 7,217) ^a^		
No	3,312	45.9
Low (dmft = 1–3)	1,948	27.0
High (dmft ≥ 4)	1,967	27.1

^a^ Total valid samples without considering the sample weights.

The prevalence of oral health impacts on daily lives, measured by the OIDP index, was 26.1% (95%CI 22.3–30.2). The questions with the highest frequencies were those related to eating (14.0%) and brushing teeth (12.4%) ( Table 2 ).

**Table 2:** Frequency distribution of the oral impacts on daily performance (OIDP) answers in five-year-old children. Brazilian Oral Health Survey, 2010.

Impaired performances because of teeth	No	95%CI	Yes	95%CI
	
	n ^a^ (%)		n ^a^ (%)	
Difficulties in eating or felt dental pain while drinking cold or hot liquids	6,268 (86.0)	82.9–88.7	986 (14.0)	11.3–17.1
Discomfort while brushing teeth	6,416 (87.6)	85.1–89.7	841 (12.4)	10.3–14.9
Teeth made child nervous or angry	6,591 (90.3)	88.2–92.1	651 (9.7)	7.9–11.8
Child avoids leisure activities	6,938 (95.1)	93.7–96.2	311 (4.9)	3.8–6.3
Child avoids sports activities	7,040 (97.2)	96.2–98.0	193 (2.8)	2.0–3.8
Difficulty speaking	6,928 (94.0)	92.2–95.4	326 (6.0)	4.6–7.8
Child feels ashamed of smiling or talking	6,891 (94.4)	92.8–95.7	372 (5.6)	4.3–7.2
Difficulty studying or doing school work	6,983 (95.9)	94.5–97.0	270 (4.1)	3.0–5.5
Difficulty sleeping	6,742 (92.0)	89.9–93.6	517 (8.0)	6.4–10.1

^a^ Total valid samples without considering the sample weights.

The results of bivariate analyses between presence of impact and the independent variables showed significant associations between the outcome and skin color/race (p = 0.015), family income (p = 0.001), dental pain (p < 0.001) and dental caries (p < 0.001) ( Table 3 ).

**Table 3:** Prevalence of oral impact on daily performances (OIDP) according to independent variables in five-year-old children. Brazilian Oral Health Survey, 2010.

Variables	Impact (OIDP)				

	No	95%CI	Yes	95%CI	p ^b^
	
	n ^a^ (%)		n ^a^ (%)		
Gender					0.372
Boys	3,673 (75.0)	75.0–79.5	882 (25.0)	20.5–30.1	
Girls	3,675 (72.8)	68.1–77.0	894 (27.2)	23.0–31.9	
Skin color/race					0.015
White	2,614 (76.5)	72.6–79.9	676 (23.5)	20.1–27.4	
Black	426 (77.4)	64.1–86.8	160 (22.6)	13.2–35.9	
*Pardo*	2,389 (70.2)	65.0–75.0	885 (29.8)	25.0–35.0	
Yellow (Asian descendants)	111 (84.5)	64.3–94.3	34 (15.5)	5.7–35.7	
Indigenous	21 (38.4)	17.1–65.2	21 (61.6)	34.8–82.9	
Family monthly income (US$)					0.001
≤ 218	1,537 (63.7)	57.2–69.8	484 (36.3)	30.2–42.8	
219–656	2,783 (72.2)	66.9–76.9	971 (27.8)	23.1–33.1	
657–1,093	845 (85.0)	77.8–90.2	189 (15.0)	9.8–22.2	
≥ 1,094	614 (84.4)	68.0–93.2	63 (15.6)	6.8–32.0	
Household overcrowding					0.955
1 room or more/person	1,355 (74.1)	68.5–79.0	336 (25.9)	21.0–31.5	
Less than 1 room/person	4,202 (73.9)	68.4–78.7	1,439 (26.1)	22.3–31.6	
Dental pain					≤ 0.001
No	4,947 (84.6)	81.2–87.5	813 (15.4)	12.5–18.8	
Yes	563 (35.0)	28.7–41.9	957 (65.0)	58.1–71.3	
Dental caries					≤ 0.001
No	2,922 (87.6)	83.8–90.6	390 (12.4)	9.4–16.2	
Low (dmft = 1–3)	1,510 (74.6)	68.5–79.9	438 (25.4)	20.1–31.5	
High (dmft ≥ 4)	1,047 (50.3)	43.0–57.6	910 (49.7)	42.4–57.0	

^a^ Corrected for sample design.

^b^ Rao-Scott test.

Values with statistical significance are shown in bold.

Table 4 shows the results of the unadjusted and adjusted Poisson regression analyses assessing the association between dental pain and the presence of impact. In the unadjusted analysis, children with dental pain (PR = 4.23, 95%CI 3.37–5.31) and those with caries experience had a higher prevalence of negative impact on quality of life. *Pardo* (PR = 1.26, 95%CI 1.06–1.51) and indigenous (PR = 2.62, 95%CI 1.71–4.00) children had a higher prevalence of impact compared to white children. Regarding family income, only those from the lowest category level had a higher prevalence (PR = 2.32, 95%CI 1.01–5.31) compared with the highest, approaching borderline significance at P = 0.047. When adjusted for the two sociodemographic variables (skin color/race and family income), dental pain and dental caries remained highly significant, although the PR estimates decreased. Children with dental pain had a higher prevalence of impact compared to those with no dental pain (PR = 1.14, 95%CI 1.06–1.23). Children with low dmft (PR 1.90, 95%CI 1.39–2.60), as well as those with high dmft (PR = 3.53, 95%CI 2.78–4.49) also had a higher prevalence of impact, compared to those with no caries experience. Family income and skin color/race were no longer associated with the outcome. The interaction between presence of dental caries and presence of pain was not significant (p = 0.989).

**Table 4:** Results of Poisson regression with robust variance analysis of the association between oral impact on daily performances (OIDP) in five-year-old children and independent variables. Brazilian Oral Health Survey, 2010.

Independent variable	Impact (no/yes)					

	Unadjusted			Adjusted		
	
	PR	95%CI	p	PR	95%CI	p
Dental pain						
No	1			1		
Yes	4.23	3.37–5.31	≤ 0.001	1.14	1.06–1.23	≤ 0.001
Skin color/race						
White	1			1		
Black	0.96	0.76–1.21	0.719	0.77	0.57–1.03	0.087
*Pardo*	1.26	1.06–1.51	0.010	1.05	0.87–1.27	0.600
Yellow (Asian descendants)	0.66	0.29–1.51	0.325	0.42	0.17–1.02	0.056
Indigenous	2.62	1.71–4.00	≤ 0.001	1.51	0.83–2.75	0.176
Family income (US$)						
≤ 218	2.32	1.01–5.31	0.047	1.57	0.70–3.51	0.267
219–656	1.78	0.77–4.11	0.178	1.32	0.59–2.97	0.491
657–1,093	0.96	0.41–2.21	0.916	0.81	0.35–1.88	0.638
≥ 1,094	1			1		
Dental caries						
No	1			1		
Low (dmft = 1–3)	2.04	1.52–2.75	≤ 0.001	1.90	1.39–2.60	≤ 0.001
High (dmft ≥ 4)	4.01	3.20–5.02	≤ 0.001	3.53	2.78–4.49	≤ 0.001

PR: prevalence ratio

## DISCUSSION

In this study, strong associations were found between dental pain as well as dental caries in five-year-old children and the impact on their daily performances, indicating that both conditions are important causes of poor quality of life in young children. These results are particularly relevant since this is the first analysis which considered OHRQoL as an outcome regarding pain and dental caries in a nationally representative sample of preschool children. They also corroborate previous investigations using other instruments developed for preschool children ^2–12,18^ .

One strength of this study is the use of a population-based sample with data collection in households, while in the previous local studies, the samples were school-based or included only children attending a national vaccination programme.

More than a quarter (26.1%) of the children were affected by their oral health condition in the six months preceding the data collection. A slightly lower frequency (21%) was reported in the UK’s Children’s Dental Health Survey 2013 ^19^ , using the same time frame. Dental pain and caries prevalences were also higher in Brazil (22% ^15^ and 54.1%, respectively) than in the UK (14% and 31%, respectively) ^19^ ^,^ ^27^ . Other studies carried out in some municipalities in Brazil and in other countries have shown varied prevalences ^3,5,6,9,11–16,18,28^ . However, comparisons with other studies are not very meaningful, since they have employed different methodologies and age ranges.

The most prevalent impaired performances were those in the physical domain: difficulties in eating and brushing teeth. Results regarding eating difficulties corroborate previous studies in preschool children ^2,4–6,18^ . Gender was not associated with impact, as reported by other researchers in Brazil and in other countries ^4^ ^,^ ^7^ ^,^ ^9^ ^,^ ^10^ .

The higher prevalence of impact among children from families with low socioeconomic condition, measured by skin color and family income, highlights the persisting inequalities in oral health in the Brazilian population. However, the association between socioeconomic status and the presence of impact was strongly influenced by dental pain and caries experience. This effect was expected since caries is the main cause of dental pain among preschool children ^13^ . A study on the same sample shows that children with caries experience had a higher prevalence of pain than caries-free individuals ^15^ ^,^ ^28^ . Regarding skin color/race, the low prevalence of indigenous children in the sample may have influenced the non-significant results after controlling for other variables.

The results in the final statistical models indicate that pain and caries may affect children’s quality of life regardless of their socioeconomic conditions, but this is not a consensus in the literature ^2,4,5,7,8–11,18^ . On the other hand, a systematic review ^29^ showed that in most of the studies including children of various ages, those from families with higher levels of income and education, as well as lower household crowding had better OHRQoL. In the present study, the lack of other important socioeconomic variables in the databank, such as parents’ level of education, may have influenced the results ^29^ .

One of the limitations of the present study is inherent to the population group investigated, since the parents’ report may not totally correspond to their children’s real experience of impact ^30^ . Due to the low cognitive capacity of children under six, a more accurate measure of their quality of life remains a challenge.

Another aspect is the instrument used to measure OHRQoL. In the 2010 Brazilian Oral Health Survey, a single questionnaire, including the conventional OIDP index, was used to measure impacts on adults, the elderly, adolescents and children aged five and 12. Although an age-specific instrument for young children is already available – the Early Childhood Oral Health Impact Scale (ECOHIS) ^31^ –, its use in large population surveys such as the Brazilian survey will not always be feasible, since the need for multiple OHRQoL instruments would result in increased time and costs. Also, the Brazilian version of the ECOHIS was not available at the time of the national survey. It is noteworthy that other studies have employed other questionnaires than ECOHIS to assess the OHRQoL of preschool children ^6^ ^,^ ^12^ . Therefore, the use of an instrument not validated for young children may be a limitation and should be considered when comparing the present results with those from previous studies using the ECOHIS ^11^ ^,^ ^12^ ^,^ ^18^ . Another methodological aspect to be discussed is the adaptation made to the original OIDP scale. Although in the present study the possible answers of the questionnaire were only ‘yes’ or ‘no’, comparisons with previous studies were possible, since they have usually dichotomized the categories in that way for statistical analysis. On the other hand, the severity of the impact could not be estimated.

Continued efforts are therefore needed to decrease the high caries prevalence and severity among young Brazilian children, which may result in fewer cavities and less suffering. Since low socioeconomic groups are at higher risk of caries, further actions to reduce social inequalities would be appropriate in order to tackle the problem. Children’s oral health may also have a negative effect on their families as well as economic impact, considering the working days lost due to caries and pain. Therefore, children’s and their families’ perception of their oral health status should be considered an important component of oral health surveillance strategies, which would help understand the impact of oral health problems on their lives, estimate the real population needs and evaluate the results of public health interventions. Comparisons between the sociodental approach, using OHRQoL instruments, and the standard normative approach, based on clinical examinations, have shown that the use of the sociodental approach results in lower estimates of needs among children ^17^ .

Finally, specific education measures are also necessary to improve families’ and health services’ awareness of the importance of primary dentition, which has generally received little attention worldwide.

It was concluded that dental pain had a strong negative impact on the daily lives of five-year-old Brazilian children and that dental caries experience was also associated with impact.
